# High-Degree Concentration Organic Solvent Forward Osmosis for Pharmaceutical Pre-Concentration

**DOI:** 10.3390/membranes14010014

**Published:** 2024-01-04

**Authors:** Ryoichi Takada, Ryosuke Takagi, Hideto Matsuyama

**Affiliations:** 1 Department of Chemical Science and Engineering, Kobe University, Kobe 657-8501, Japan; takada.rg@om.asahi-kasei.co.jp; 2 Asahi Kasei Corporation, Chiyoda-Ku, Tokyo 100-0006, Japan; 3 Research Center for Membrane and Film Technology, Kobe University, Kobe 657-8501, Japan; takagi@harbor.kobe-u.ac.jp

**Keywords:** organic solvent forward osmosis, high-degree concentration, active pharmaceutical ingredients, polyketone-based thin-film composite hollow fiber membrane

## Abstract

Over half of the pharmaceutical industry’s capital investments are related to the purification of active pharmaceutical ingredients (APIs). Thus, a cost-effective purification process with a highly concentrated solution is urgently required. In addition, the purification process should be nonthermal because most APIs and their intermediates are temperature-sensitive. This study investigated a high-degree concentration organic solvent forward osmosis (OSFO) membrane process. A polyketone-based thin-film composite hollow fiber membrane with a polyamide selective layer on the bore surface was used as the OSFO membrane to achieve a high tolerance for organic solvents and an effective concentration. MeOH, sucrose octaacetate (SoA), and 2M polyethylene glycol 400 (PEG-400)/MeOH solution were used as the solvent, model API, and a draw solution (DS), respectively. OSFO was performed at room temperature (23 ± 3 °C). Consequently, the 11 wt% SoA/MeOH solution was concentrated to 52 wt% without any SoA leakage into the DS. To our knowledge, there are no studies in which up to a 5 wt% concentration by OSFO has been demonstrated. However, the final feed solution contained 17 wt% PEG-400. This study demonstrates the promising potential of OSFO for pharmaceutical pre-concentration and the technical problems that need to be solved for social implementation.

## 1. Introduction

Every year, the pharmaceutical industry develops new drugs to improve the quality of life. In 2021, the U.S. Food and Drug Administration (FDA) approved 50 new drugs [1]. Although protein- and nucleotide-based drugs have recently attracted attention, more than half of the new drugs are small-molecule-based [1]. The active ingredients (active pharmaceutical ingredients, APIs) of small-molecule-based drugs are typically manufactured by multi-step chemical synthesis. At each step, the APIs and their intermediates are purified and isolated for quality check. However, the purification and isolation processes are costly. More than half of the capital investment in this area is related to purification and isolation processes [2]. Therefore, cost-effective purification and isolation processes are urgently needed for drug development. 

One of the promising ways to reduce the cost drastically is the pre-concentration of the process solutions before the purification and isolation process. The process solutions are solutions at intermediate stages in the manufacture of pharmaceuticals. The reducing volume of the process solution results in a smaller capacity and shortens the time required in the subsequent process. In general, the capital investment of a plant roughly decreases in proportion to the power of 0.6 of its capacity (the rule of six-tenths) [3], and shorter process time may decrease the operation cost. Thus, the pre-concentration of the process solution may reduce the purification and isolation cost drastically.

The promising pre-concentration process has several requirements. First, the capital investment of the pre-concentration process and the cost-benefit in the following processes must be balanced. Both the small capital cost of the pre-concentration process and the high-degree concentration ratio can open up opportunities for this concept. Second, the pre-concentration process should be a non-thermal process because most APIs and their intermediates are temperature-sensitive [4].

Given these requirements, the organic solvent forward osmosis (OSFO) is a potential process for pre-concentration. OSFO is a membrane-based technology that moves solvent from a low to a high osmotic pressure side, i.e., from a feed solution (FS) to a draw solution (DS). Theoretically, OSFO can concentrate FS to any concentration if there is an osmotic pressure difference between FS and DS. Therefore, a high-degree concentration of process liquid can be achieved by selecting an appropriate DS. In addition, no external pressure is needed in OSFO, which is an excellent advantage in capital cost against pressure-driven processes, such as organic solvent nanofiltration (OSN). Although the OSFO has disadvantages such as low solvent flux and the necessity of the regeneration of DS, a high-degree concentration with lower capital cost that could be achieved by OSFO may more than compensate for these disadvantages. Moreover, OSFO does not require a thermal input, which can minimize the degradation of APIs and their intermediates during concentration.

Several groups have demonstrated OSFO concentrations in the last decade. In these reports, LiCl, methyl palmitate, citric acid, PEG-400, PEG-1000, and diethanolamine were shown as workable draw solutes in OSFO [4,5]. Flat sheet membranes or hollow fiber (HF) membranes were used [4,5]. The HF-type membrane is advantageous for OSFO because it can generate an appropriate draw solution flow. Furthermore, an HF-type membrane with a selective layer on the bore surface will be the best configuration for high-degree concentrations of APIs. This can help minimize the hold-up volume of the concentration system, resulting in a secure collection of valuable APIs.

Recently, Goh et al. developed a thin-film composite (TFC) membrane with a polyamide-selective layer on the bore surface of a crosslinked polyimide HF and demonstrated a model case of pharmaceutical concentration [5]. OSFO successfully concentrated levofloxacin in acetone to 16,000 ppm (ca. 2 wt% calculated from the density of acetone). However, considering that solutions above 5 wt% are typically used in practical applications [6], OSFO pre-concentration should concentrate solutions from at least 5 wt% to a higher concentration to show the effect. Cui et al. confirmed a flux of 1.35 L m^−2^ h^−1^ in the system where 20% triglycerides/hexane was used as a feed solution and 50% methyl palmitate/hexane solution as a DS [4]. This experiment successfully shows the possibility of a high-degree concentration of up to 20% of feed solution concentration, but it was just a solvent recovery, and no concentration behavior of API was shown. Despite several OSFO demonstrations, to our knowledge, suitable studies that can prove the concept of OSFO pre-concentration have yet to be demonstrated.

In this study, we developed a TFC hollow fiber membrane with a polyamide-selective layer on the bore surface using polyketone porous HF as a support. Since the high tolerance of substrate to organic solvents is one of the key properties required for OSFO membrane, we employed polyketone porous HF as a substrate. With this solvent-resistant HF membrane, we attempted to develop an OSFO system that concentrated APIs from more than 5 wt% to higher concentrations.

## 2. Experimental Section

### 2.1. Materials and Chemicals

Asahi Kasei Corporation, Japan, kindly provided a polyketone porous HF as a commercially unavailable sample. It was used as an HF support for the TFC membrane. For the interfacial polymerization reaction, 1,3-phenylenediamine (MPD), sodium dodecyl sulfate (SDS), and hexane were obtained from FUJIFILM Wako Pure Chemical Co., Ltd., Osaka, Japan, and 1,3,5-benzenetricarbonyl trichloride (TMC) was obtained from Tokyo Chemical Industry, Japan. For the evaluation of membrane quality, MgSO_4_ (FUJIFILM Wako Pure Chemical Co.) and Brilliant Blue R (BBR) (Tokyo Chemical Industry, Tokyo, Japan) were used. D-(+)-sucrose octaacetate (SoA) (Tokyo Chemical Industry, Japan) was used as a model API because of its high solubility in various organic solvents and suitable molecular weight (679 Da). Polyethylene glycol 400 (PEG-400) (FUJIFILM Wako Pure Chemical Co.) was used as a draw solute because of its low toxicity and very high osmotic pressure [7]. Among the commercially available PEG, PEG-400 is liquid at room temperature. Therefore, highly concentrated solutions can be easily prepared. MeOH was selected as a solvent of feed and draw solutions since it is one of the preferred solvents in medicinal process chemistry [8,9].

### 2.2. Characterization of HF Support

#### 2.2.1. Pore Size and Porosity

The mean pore size of the polyketone HF support was measured using a capillary flow porometer (CFP-1200AX; Porous Materials, Inc., Ithaca, NY, USA) according to the Japanese Industrial Standards (JIS) K3832 [10] as shown in the supplemental file (Figure S1). 

The porosity of the polyketone HF support was measured by a gravimetric method [11]. The weights of 10 pieces of 10-cm-long HF were measured using an electronic balance. The porosity of HF, *ϕ* (%), was obtained by Equation (1), where *W* (g) was the measured weight of the HFs; *ID* (cm) and *OD* (cm) were the inner and outer diameters of the HF, respectively; and *ρ_PK_* the density (g/cm^3^) of the polyketone, which was taken as 1.3 g/cm^3^ [12].

(1) $$\phi =100\times \left[1-\frac{W}{10}\left\{\frac{4}{\rho_{PK}\times 10\times \left(OD^{2}-ID^{2}\right)\times \pi }\right\}\right]$$

#### 2.2.2. Water Permeation of Support

**Figure 1:**
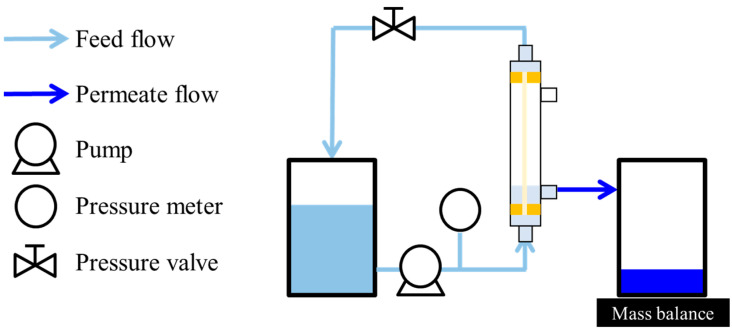
Experimental setup for permeation measurement.

The water permeation of the HF support was measured by a pressure-driven permeation experiment using a single laboratory-scale HF module, which contains a single HF with an effective length of 12.5 cm. Figure 1 shows the setup of the laboratory-scale permeation measurement. The flow rate and transmembrane pressure were set to 10 mL/min (0.89 m/s) and 1.00 bar (0.100 MPa), respectively. Deionized (DI) water was used as the feed solution for the permeation measurement. The permeation measurement was conducted at room temperature (23 ± 3 °C). The permeates collected for at least 2 min. The water permeance, *J_w_* (L m^−2^ h^−1^ bar^−1^), was given by Equation (2), where *m_w_* (kg) was the mass of water collected, Δ*P* (bar) was the transmembrane pressure, *A* (m^2^) was the effective membrane area based on the bore surface of HF support (0.00019 m^2^), *ρ_w_* (kg L^−1^) was the density of water and put as 1.000, and *t* (h) was the time for permeate collection.

(2) $$J_{w}=\frac{m_{w}}{\Delta P\times A\times \rho_{w}\times t}$$

### 2.3. Fabrication of TFC-HF Membrane

Since active layer formed by interfacial polymerization from MPD and TMC was reported to reject small molecules above 300 Da in molecular weight [13], we selected the same active layer material to prove the OSFO pre-concentration concept.

**Figure 2:**
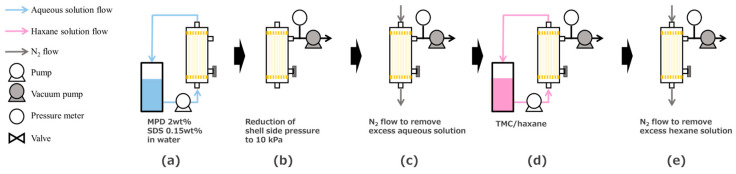
Experimental setup for interfacial polymerization; (**a**) Circulation of aqueous solution, (**b**) Reduction of shell side pressure, (**c**) N_2_ flow to remove excess solution, (**d**) Circulation of hexane solution and (**e**) N_2_ flow to remove excess solution.

The TFC-HF membrane was fabricated using a laboratory-scale HF module which contains 80 pieces of HF with an effective length of 8.0 cm as described in our patent application [14]. The module was set into an apparatus shown in Figure 2, and the vacuum-assisted interfacial polymerization was performed [14,15,16]. An aqueous solution containing 2 wt% MPD and 0.15 wt% SDS was circulated through the bore side of the HF support for 6 min (Figure 2a). The module was allowed to stand for 5 min. At this time, the shell side was kept open to the air. Then, the shell-side pressure was decompressed to 10 kPa (Figure 2b). Subsequently, the excess aqueous solution on the bore surface was removed by an N_2_ gas flow (Figure 2c). Then, while maintaining the shell-side pressure, a hexane solution containing 0.2 wt% TMC was introduced into the bore side of the HF support (Figure 2d). The HF supports were left to react for 1 min, and the excess hexane solution was blown out using N_2_ gas (Figure 2e). Finally, the shell-side pressure was relieved, and the module was heat-treated at 50 °C for 5 min. The final TFC-HF module was washed at least overnight with a large amount of deionized (DI) water and stored in DI water before use.

### 2.4. Characterization of TFC-HF Membrane

#### 2.4.1. SEM Observation

The cross-sectional and surface morphologies of the HF and TFC-HF membranes were observed by scanning electron microscopy (SEM; FlexSEM1000, Hitachi High-Tech Co., Ltd., Tokyo, Japan). The HF samples were frozen and fractured in liquid nitrogen and then coated with osmium tetroxide (OsO_4_) using an osmium coater (HPC-1SW; VACUUM DEVICE Co., Ltd., Mito, Japan) for SEM observation.

#### 2.4.2. XPS Analysis

The HF support and TFC-HF membrane bore surfaces were examined by X-ray photoelectron spectroscopy (XPS; PHI VersaProbe 4, ULVAC-PHI, Inc., Chigasaki, Japan) to confirm the polyamide active layer. The HF support and TFC-HF membrane were cut to expose the bore surface, and XPS measurements were performed.

#### 2.4.3. Attenuated Total Reflection Fourier Transform Infrared Spectroscopy (ATR-FTIR)

The HF support and TFC-HF membrane bore surfaces were examined by ATR-FTIR (FT/IR-6100, JASCO corporation, Tokyo, Japan) to confirm the formation of a polyamide active layer. The HF support and TFC-HF membrane were cut to expose the bore surface, and ATR-FTIR measurements were performed.

### 2.5. Performance of TFC-HF Membrane

#### 2.5.1. Rejection Performance under Pressurized Condition

The rejection performance was evaluated using the experimental setup shown in Figure 1, except for the module used. The module used in this experiment was prepared in Section 2.3. The flow rate of the feed solution was 36 mL/min (0.040 m/s), and the transmembrane pressure was 2.00 bar (0.200 MPa). A 0.1 wt% (0.0083 mol L^−1^) MgSO_4_ aqueous solution or 0.005 wt% (6 × 10^−5^ mol L^−1^) BBR/MeOH solution was used as the feed solution. The experiment was carried out at room temperature (23 ± 3 °C) for at least two modules. The permeates collected for at least 2 min after at least 30 min of conditioning. The solvent permeance, *Ji* (L m^−2^ h^−1^ bar^−1^), was given by Equation (3), where the subscript *i* indicated water or MeOH, *m_i_* (kg) the mass of permeate collected, Δ*P* (bar) the transmembrane pressure, Δ*π* (bar) is the difference of osmotic pressure between the feed solution and the permeate, *A* (m^2^) the effective surface area of the module based on the bore side (0.010 m^2^), *ρ_i_* (kg L^−1^) the density of permeate, and *t* (h) the time for permeate collection. The osmotic pressure (bar) is given by the Van ’t Hoff equation (Equation (4)) where *i* is the dimensionless Van ’t Hoff factor, *c* (mol L^−1^) is the molar concentration of solute, *R* (L bar K^−1^ mol^−1^) is the ideal gas constant, and *T* (K) is the absolute temperature.

(3) $$J_{i}=\frac{m_{i}}{\left(\Delta P-\Delta \pi \right)\times A\times \rho_{i}\times t}$$

(4) π=icRT

In the calculation of permeance, it is reasonable to consider the osmotic pressure of permeate negligibly small since the apparent rejection is nearly 100 % for MgSO_4_ and BBR. 

Thus, the osmotic pressure of feed was used as Δ*π* in Equation (3). Additionally, 0.41 bar was obtained as the osmotic pressure of 0.1 wt% MgSO_4_ aqueous solution by substituting 2, 0.0083 mol L^−1^, 0.083 L bar K^−1^ mol^−1^, and 298.15 K into *i*, *c*, *R* and *T*, respectively. The osmotic pressure of the 0.005 wt% BBR/MeOH solution was negligibly small compared with the operating pressure 2 bar. As the density of permeate, 1.000 (kg L^−1^) was used for water, and 0.792 (kg L^−1^) [17] was used for MeOH.

The apparent rejection of the solute *R_j_* (%) was given by Equation (5), where the subscript *j* indicated MgSO_4_ or BBR. *C_jf_* (wt%) and *C_jp_* (wt%) were the concentrations of solute in the feed and permeate, respectively. The MgSO_4_ concentration was determined from the conductivity, which was measured using a conductivity meter (ECTESTR 11+; Thermo Scientific Eutech Instruments, Landsmeer, The Netherlands). BBR concentration was determined from the absorbance at 588 nm using a UV-Vis spectrometer (UV-2400PC, Shimadzu, Japan).

(5) $$R_{j}=\frac{C_{jf}-C_{jp}}{C_{jf}}\times 100$$

#### 2.5.2. OSFO Performance

**Figure 3:**
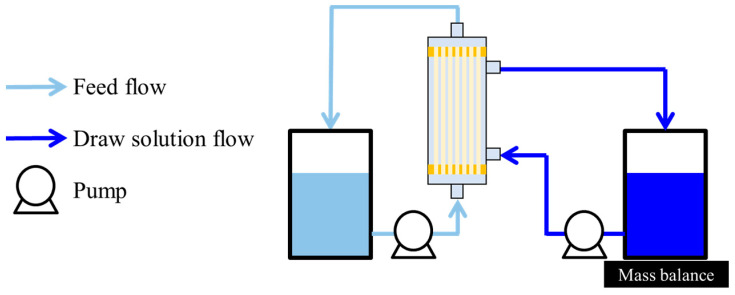
Experimental setup for OSFO.

The MeOH flux was measured to evaluate the OSFO performance using the experimental setup shown in Figure 3. A sucrose octaacetate (SoA)/MeOH solution and a 2 M PEG-400/MeOH solution were used as the feed solution and the draw solution, respectively. The measurement was carried out at various SoA concentrations (0–60 wt%) using a single module. FS and DS were replaced before each measurement. The operation time, flow rate of the feed solution and the draw solution, initial FS amount, and initial DS amount were fixed to 20 min, 36 mL/min (0.040 m/s), 336 mL/min (0.025 m/s), 150 g and 1000 g, respectively. The measurement was carried out at room temperature (23 ± 3 °C) for at least two modules to confirm the reproducibility.

The MeOH flux, *J_MeOH_* (L m^−2^ h^−1^), was given by Equation (6), where Δ*m_d_* (kg) was the change in mass of draw solution over a time interval Δ*t* (h), and Δ*m_PEGf_* (kg) the change in mass of PEG-400 in feed solution over a time interval Δ*t* (h). *A* (m^2^) was the effective area of the bore surface of the module (0.010 m^2^). *ρ_MeOH_* (kg L^−1^) was the density of MeOH (0.792 kg L^−1^) and *ρ_PEG_* (kg L^−1^) the density of PEG-400 (1.13 kg/L) [18]. The concentration of PEG-400 was measured using LC/MS (LC; ACQUITY UPLC I-Class, Nihon Waters Co., Ltd., Tokyo, Japan/MS; micrOTOF-QIII, Bruker Daltonics, Billerica, MA, USA).

(6) $$J_{MeOH}=\frac{\Delta m_{d}/\rho_{MeOH}+\Delta m_{PEGf}/\rho_{PEG}}{A\times \Delta t}$$

#### 2.5.3. High-Degree Concentration by OSFO

A high-degree concentration by OSFO was conducted using the experimental setup shown in Figure 3. In this experiment, the initial SoA concentration was set at approximately 11 wt%. The other experimental conditions were the same as those described in the previous section, except for the long operation time. The experiment was carried out at room temperature (23 ± 3 °C) for at least two modules to confirm the reproducibility. The MeOH flux was given by Equation (6). The PEG-400 and SoA concentrations were measured using LC/MS (LC; ACQUITY UPLC I-Class, Nihon Waters Co., Ltd., Tokyo, Japan/MS; micrOTOF-QIII, Bruker Daltonics, Billerica, MA, USA). Concentration ratio was calculated by Equation (7), where *V(t)* (mL) and *V_initial_* (mL) were the volume of feed solution at given operation time and the volume of initial feed solution, respectively.

(7) $$Concentration\;ratio=\frac{V\left(t\right)}{V_{initial}}$$

## 3. Results and Discussion

### 3.1. Characterization of HF Support

**Figure 4:**
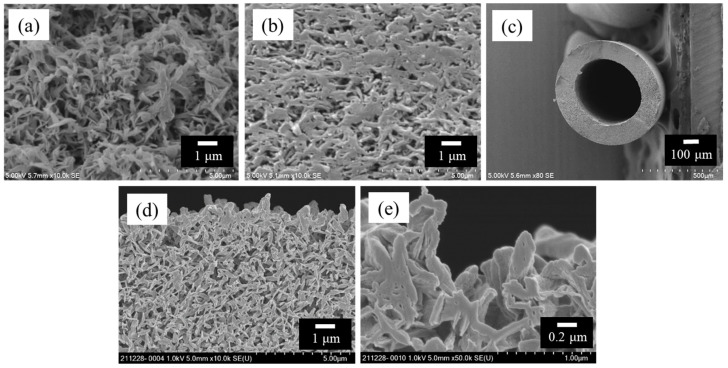
SEM images of polyketone HF support; (**a**) bore surface, (**b**) shell surface, (**c**) cross-section with low magnification, (**d**) cross-section near the bore surface and (**e**) cross-section near the bore surface with high magnification.

The characteristics of the HF supports are summarized in Table 1. In this paper, the error shown in the table and figure is the probable error. Figure 4 shows the SEM images of the surfaces and cross-sections of the HF support. As shown in Figure 4a, the bore surface of the HF support had an open-pore and nonwoven fabric-like morphology. These surface morphologies of the HF support were similar to those of the previously fabricated flat sheet PK support [19].

In this study, the inner diameter of the HF was 488 μm as shown in Table 1, and we used a TFC-HF membrane with a 0.010 m^2^ effective bore surface. Thus, the effective surface area corresponds to about 1.2 mL inner volume. This means that the smallest volume of feed solution that our HF system can treat is 1.2 mL. On the other hand, putting a spacer thickness in a flat sheet membrane system of 28 mil (0.84 mm) [20], 0.010 m^2^ of flat sheet membrane module corresponds to 4.2 mL inner volume. Considering that both systems have the same concentration performance, it means that our HF system can concentrate a feed solution 3.5 times higher than a flat sheet membrane system since the smallest volume that our HF system can treat is 3.5 times smaller than that of the flat sheet membrane system. This is the reason why the HF membrane with the selective layer on the bore surface is selected as OSFO membrane.

### 3.2. Characterization of the Selective Layer

**Figure 5:**
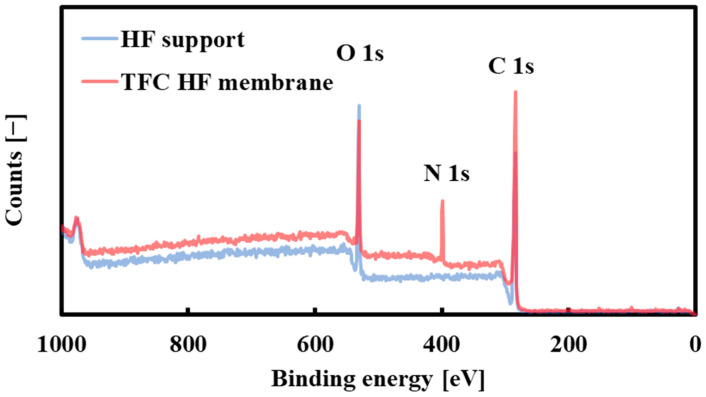
X-ray photoelectron spectra of the bore surface before (HF support) and after (TFC-HF membrane) the interfacial polymerization.

Figure 5 shows the bore surface’s X-ray photoelectron spectra (XPS) before and after interfacial polymerization. It is clear from Figure 5 that an N atom peak originating from the aromatic polyamide active layer appears at 400 eV after interfacial polymerization. These data are the same as the XPS data reported in the literature [21] in which the aromatic polyamide active layer was formed on the shell surface of the polyketone support (Figure S2). It shows that a polyamide-selective layer was successfully formed on the bore surface of the HF support. 

**Figure 6:**
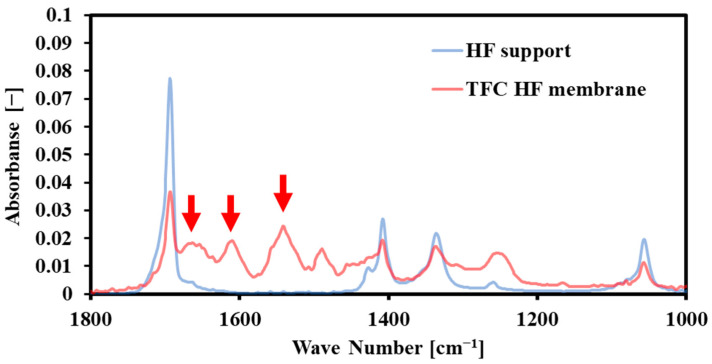
ATR-FTIR of the bore surface before (HF support) and after the interfacial polymerization (TFC-HF membrane). (Arrows indicated the peaks at 1541, 1610, and 1663 cm^−1^, respectively).

Figure 6 shows the ATR-FTIR spectra of the bore surface before and after interfacial polymerization. The spectrum of HF support showed four peaks at 1057, 1336, 1408, and 1693 cm^−1^. These peaks were also identified in the TFC-HF membrane due to the high penetration depth of the IR beam (>300 nm) at this region (1000–1800 cm^−1^). In addition, the three new peaks at 1541, 1610, 1663 cm^−1^ in the spectrum of the TFC-HF membranes can be assigned to amide II band (N-H in-plane bending and N-C stretching vibration of a -CO-NH- group), aromatic amide (N-H deformation vibration) and amide I band (C=O stretching-dominant contributor, C-N stretching, and C-C-N deformation vibration in a secondary amide group), respectively [21]. These peaks revealed the presence of an interfacially polymerized polyamide layer at the bore surface.

**Figure 7:**
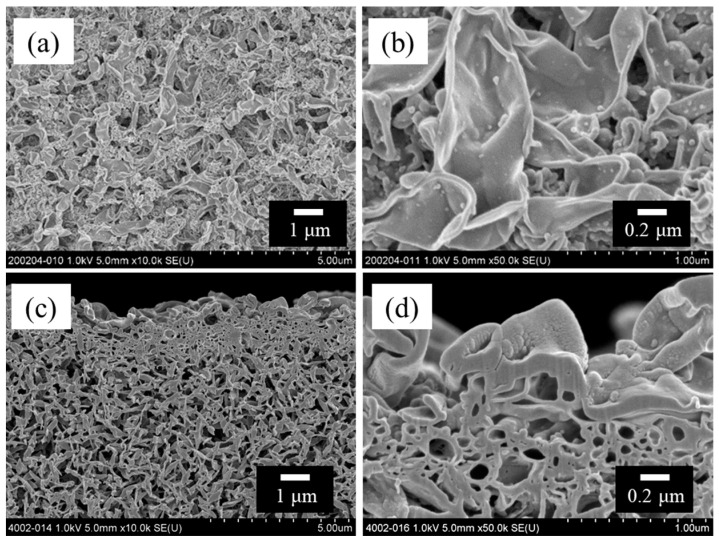
SEM images of TFC-HF membrane; (**a**) bore surface, (**b**) bore surface with high magnification, (**c**) cross-section near the bore surface, and (**d**) cross-section near the bore surface with high magnification.

Figure 7 shows SEM images of the bore surface of the TFC-HF membrane. The typical “ridge and valley” structure of polyamide selective layer is found on the HF support’s bore surface. 

### 3.3. Apparent Rejection of the TFC-HF Membrane

**Figure 8:**
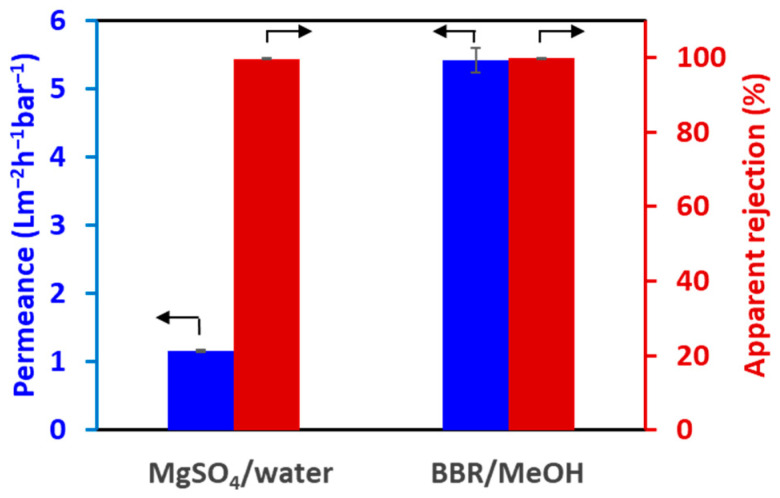
Performance of the TFC-HF membrane under pressure-driven process.

Because APIs are valuable, it is important that the selective layer is defect-free to avoid any loss of APIs. A pressure-driven permeation experiment was conducted to confirm the formation of a defect-free selective layer via interfacial polymerization, as described in Section 2.5.1. MgSO_4_ was used as the marker, and DI water was used as the solvent. The apparent rejection of MgSO_4_ was nearly 100 % (Figure 8), indicating the successful formation of a defect-free selective layer.

A similar experiment was conducted to confirm whether the defect-free selective layer can reject a small molecule in organic solvents. BBR and MeOH were used as markers and solvents, respectively. The apparent rejection of BBR was nearly 100% (Figure 8), confirming that the fabricated TFC-HF membrane had sufficient tolerance for MeOH.

The flux of MeOH was much higher than that of water. This is probably because of the lower viscosity of MeOH and the swelling of the polyamide selective layer with MeOH.

### 3.4. OSFO Performance of the TFC-HF Membrane

**Figure 9:**
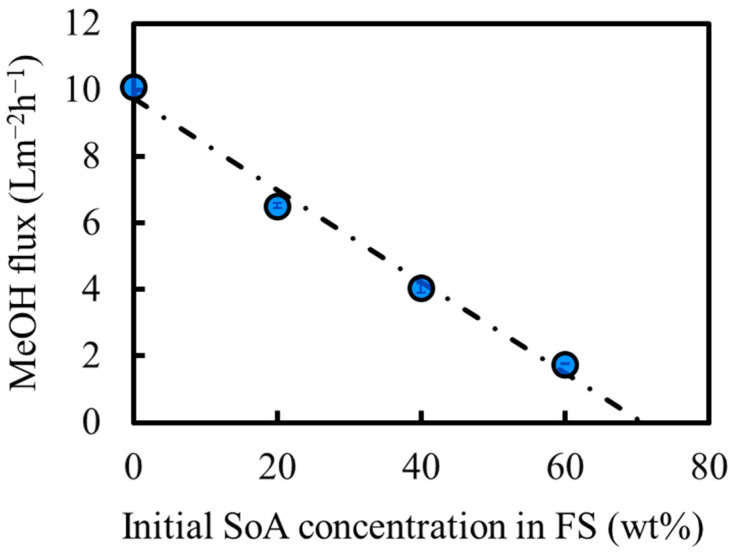
Effect of initial feed concentration on solvent flux under OSFO operation.

The OSFO performance was evaluated as a function of feed concentration by the method described in Section 2.5.2. A MeOH solution of SoA was used as the feed, and 2 M PEG-400/MeOH solution was used as the draw solution as explained in Section 2.1. The effective surface area based on the bore surface was 0.010 m^2^. Figure 9 shows the MeOH flux of OSFO as a function of SoA concentration in the feed solution. The MeOH flux decreased with increasing SoA concentration. This was because the osmotic pressure of the feed increased with increasing SoA concentration, resulting in a decrease in the osmotic pressure difference between the feed and draw solutions, which acted as a driving force for the MeOH flux. Nevertheless, there was still MeOH flux at 60 wt% SoA. Note that the flux decreased in a linear manner. If there is severe SoA concentration polarization on the bore surface, the flux is supposed to decrease more drastically with the increase of the concentration of SoA. This may show that the FS flow rate (0.040 m/s) was enough to avoid SoA concentration polarization to a practical extent. From Figure 9, it is expected that a solution containing up to 71 wt% SoA can be concentrated using our OSFO system.

### 3.5. High-Degree Concentration of the Model Solution

In the previous section, we confirmed that our OSFO system could concentrate APIs up to 71 wt%. Therefore, we attempted a high-degree concentration of the model solution using our OSFO system by the method described in Section 2.5.3. To our knowledge, the previously reported concentrations are up to 16,000 ppm by OSFO [5] and up to 20 wt% by OSN [22]. In this section, the OSFO concentration starts from approximately 11 wt% of the SoA methanol solution as the feed solution. The other experimental conditions were the same as those in Section 3.4 except for the long operation time.

**Figure 10:**
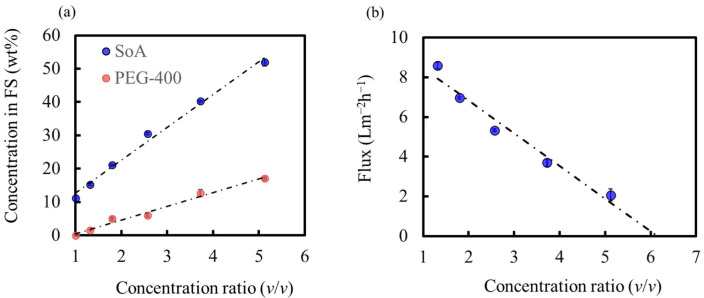
(**a**) Concentration of SoA and PEG-400 in FS during operation (**b**) MeOH Flux (L m^−2^ h^−1^) during operation.

**Figure 11:**
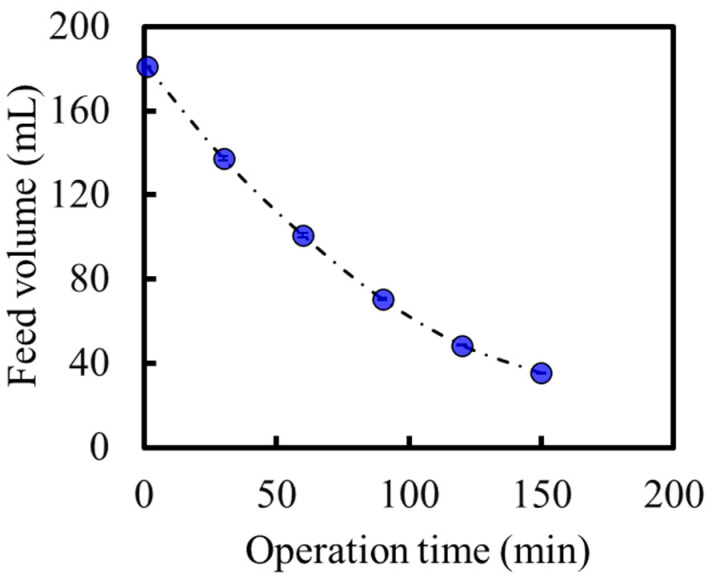
The time course of feed volume (mL).

Figure 10a shows the SoA and PEG-400 concentrations of the feed solution as a function of the concentration ratio (Equation (7)). SoA was successfully concentrated to 52 wt%, which has never been reported for API solution concentrations based on membrane technology. The SoA concentration in the final draw solution was not detected (below the detection limit), indicating that our OSFO system concentrated the API solution without SoA leakage. One of the reasons for achieving such a high-degree concentration is that the feed solution flowed through the bore side of the HF membrane in our system. Thus, the hold-up volume of the OSFO is kept very low, and it is possible to treat a relatively small volume of feed compared with the effective membrane surface. In this case, the hold-up volume was approximately 1.2 mL, whereas the effective membrane was 0.010 m^2^. In addition, a high concentration resulted in a significantly reduced feed volume. In our case, the feed volume was reduced from approximately 181 to 35 mL, as shown in Figure 11. In the purification process of the pharmaceutical industry, a small volume of processed liquid, as well as a high-degree concentration, is very important to reduce the required capacity and process time of the following process. Thus, our OSFO process satisfies these two critical points.

However, the concentration of PEG-400 in the feed solution also increased to 17 wt% in the final feed solution, as shown in Figure 10a. This PEG-400 concentration in the feed was far from acceptable according to the pharmaceutical standard, even though the SoA concentration was 52 wt%. The final feed solution of 100 g contained 52 g SoA and 17 g PEG-400. Even when all MeOH was removed, the SoA concentration was (52/(52 + 17) = ) 75 wt%. The API’s or its intermediate’s purity in pharmaceutical processes must reach more than 94 wt% [9]. Thus, the purity of SoA must be improved in later stages (e.g., crystallization). The increase in the PEG-400 concentration in the feed was due to the reverse flux of PEG-400 from the draw solution to the feed solution. There are many inhibition strategies for reverse flux of draw solute in FO, including draw solute development, membrane development, and modified operation conditions [23]. Although they are for the water system, some of the strategies may apply to improve the OSFO system. Further studies should be focused on the reverse flux of draw solute inhibition during pre-concentration.

Regarding the concentration process, the increase in the SoA concentration in the feed was due to MeOH permeation from the feed to the draw solution. This indicated a decrease in the PEG-400 concentration in the draw solution with an increase in the SoA concentration in the feed. The reduction in the PEG-400 concentration in the draw solution was also accelerated by the reverse flux of PEG-400, as shown in Figure 10a. The decrease in the PEG-400 concentration in the draw solution indicates a decrease in the osmotic pressure of the draw solution. In contrast, the increase in the SoA and PEG-400 concentrations in the feed indicates an increase in the osmotic pressure of the feed. Thus, the osmotic pressure difference between the feed and draw solutions decreased with increasing SoA concentration in the feed. Subsequently, the MeOH flux decreased with concentration (i.e., with concentration ratio), as shown in Figure 10b. According to Figure 10b, the MeOH flux will be zero at approximately 6.1 of the concentration ratio, and the concentration will stop at this point. At this point, the SoA concentration in the feed will be about 63 wt%, and lower than 71 wt%, which is the limit shown in Figure 9. As discussed above, the PEG-400 concentration in the draw solution decreases with time, while in Figure 9, the PEG-400 concentration in the draw solution is constant. Thus, it is reasonable that the maximum concentration in this experiment will be lower than that shown in Figure 9.

With a decrease in the MeOH flux, it is assumed that the concentration rate of SoA decreases simultaneously. Nevertheless, as shown in Figure 10a, the concentration rate (gradient of the SoA concentration curve) did not show a tendency to decrease with concentration. This is because the feed volume decreases simultaneously, as shown in Figure 11. Thus, the concentration rate of SoA in the feed did not show a tendency to decrease even though the MeOH flux decreased because the feed volume decreased simultaneously. However, the concentration stops when the osmotic pressure of the feed becomes the same as that of the draw solution. 

In addition, Figure 11 indicates that our membrane is possible to treat a feed for 150 min. In the pharmaceutical process, filters are typically single-use [24,25]. So, in real-world conditions, several hours of durability are enough for practical use.

## 4. Conclusions

This study developed a polyketone-based HF-type TFC membrane with a selective layer on the bore surface for OSFO because the membrane must have a high tolerance to organic solvents. Then, a high-degree concentration of API by OSFO was attempted using an SoA methanol solution as a model API solution and 2M PEG-400 methanol solution as a draw solution. As a result, SoA was successfully concentrated without any leakage loss, and a 52 wt% SoA solution was obtained. Such high-degree concentrations by OSFO or OSN have yet to be reported in API solutions thus far. This is because the hold-up volume of the OSFO system can be designed to be very small. After all, the membrane has a selective layer on its bore surface, resulting in a secure collection of concentrated solutions containing valuable APIs.

While the high-degree concentration that was the mandatory requirement for this research was achieved for the first time, the severe reverse flux of draw solute took place. The final feed solution contained 17 wt% PEG-400, which is far from acceptable by pharmaceutical standards. Thus, a purity upgrade at a later stage is necessary for the current OSFO system.

This study showed the potential of OSFO for pharmaceutical concentration and the technical problems to be solved in the future for social implementation. The reverse flux of draw solute is the next problem that should be fixed to improve the pre-concentration processes. For this problem, the strategies attempted in the water system, such as draw solute development, membrane development and modified operation conditions, will be of some help [23].

## Figures and Tables

**Table 1:** HF support characteristics. Error is the probable error.

	HF Support
Inner diameter (μm)	488 ± 5
Outer diameter (μm)	784 ± 3
Thickness (μm)	148 ± 3
Porosity (%)	74.3 ± 0.2
Mean pore size (nm)	110 ± 4
Water permeance (L m^−2^ h^−1^ bar^−1^)	2.21 ± 0.05 × 10^3^

## Data Availability

The data is contained within the article and Supplementary Materials.

## Supplementary Materials

The following supporting information can be downloaded at: https://www.mdpi.com/article/10.3390/membranes14010014/s1, Figure S1: (a) X-ray photoelectron spectra of the bore surface before (HF support) and after (TFC-HF membrane) the interfacial polymerization (this work). (b) X-ray photoelectron spectra of the shell surface before (HF-A) and after (TFC-FO) the interfacial polymerization in the literature [11]; Figure S2: Example set of data of pore size measurement.
