# Supplementary material for: High-Degree Concentration Organic Solvent Forward Osmosis for Pharmaceutical Pre-Concentration
Source: Membranes (Basel). 2024 Jan 4;14(1):14. doi: 10.3390/membranes14010014 (PMC10819892; doi:10.3390/membranes14010014)
Supplement: Supplementary file 1 [file membranes-14-00014-s001.zip › membranes-2712524-supplementary.pdf]

Supplementary Information

High-concentration by organic solvent forward osmosis for pharmaceutical pre-concentration

Ryoichi Takada [1,2], Ryosuke Takagi [3] and Hideto Matsuyama [1,3,*]

[1] Department of Chemical Science and Engineering, Kobe University, Kobe 657-8501, Japan; takada.rg@om.asahi-kasei.co.jp
[2] Asahi Kasei Corporation, Chiyoda-Ku, Tokyo 100-0006, Japan
[3] Research Center for Membrane and Film Technology, Kobe University, Kobe 657-8501, Japan; takagi@harbor.kobe-u.ac.jp
* Correspondence: matuyama@kobe-u.ac.jp; Tel.: +81-78-803-6180

1. Pore size and porosity

The mean pore size of the polyketone HF support was measured using a capillary flow porometer (CFP-1200AX; Porous Materials, Inc., USA) according to the Japanese Industrial Standards (JIS) K3832 [1] standard testing method for bubble points in membrane filters. The HF sample was first set in the machine, and pressure was applied to the bore side using dry air. In this measurement, the flow rate increased with the pressure, and a dry curve was obtained, as shown in Fig. 1. A half-dry curve was obtained by halving the dry curve. Then, the HF sample was soaked in Galwick®, whose surface tension was 15.9 dynes/cm, resulting in a wet sample. A wet sample was also set in the machine, and a wet curve was obtained. The mean pore size of the HF support, D_{pore} (μm), was given by Eq. (1), where P (psi) was the mean flow pore pressure which was obtained from the intersection of the wet curve and the half-dry curve, γ (dynes cm^{-1}) the surface tension of Galwick®, and θ the contact angle of Galwick® on polyketone which was zero in this case.

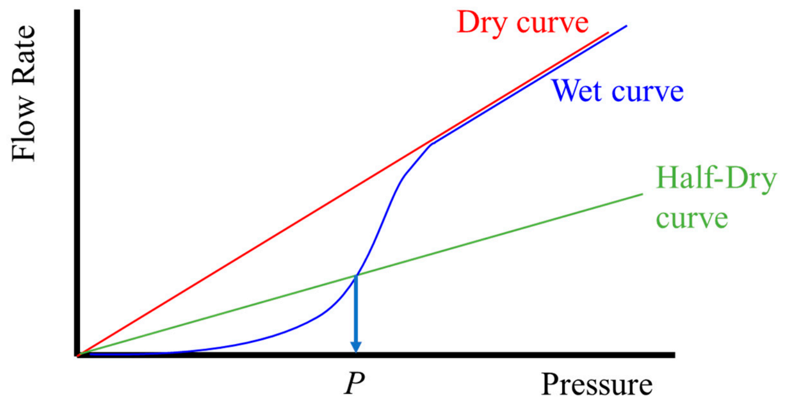

Figure S1. Example set of data.

$$D_{pore} = \frac{4\gamma cos\theta}{P} \quad (S1)$$

2. Comparison of X-ray photoelectron spectra

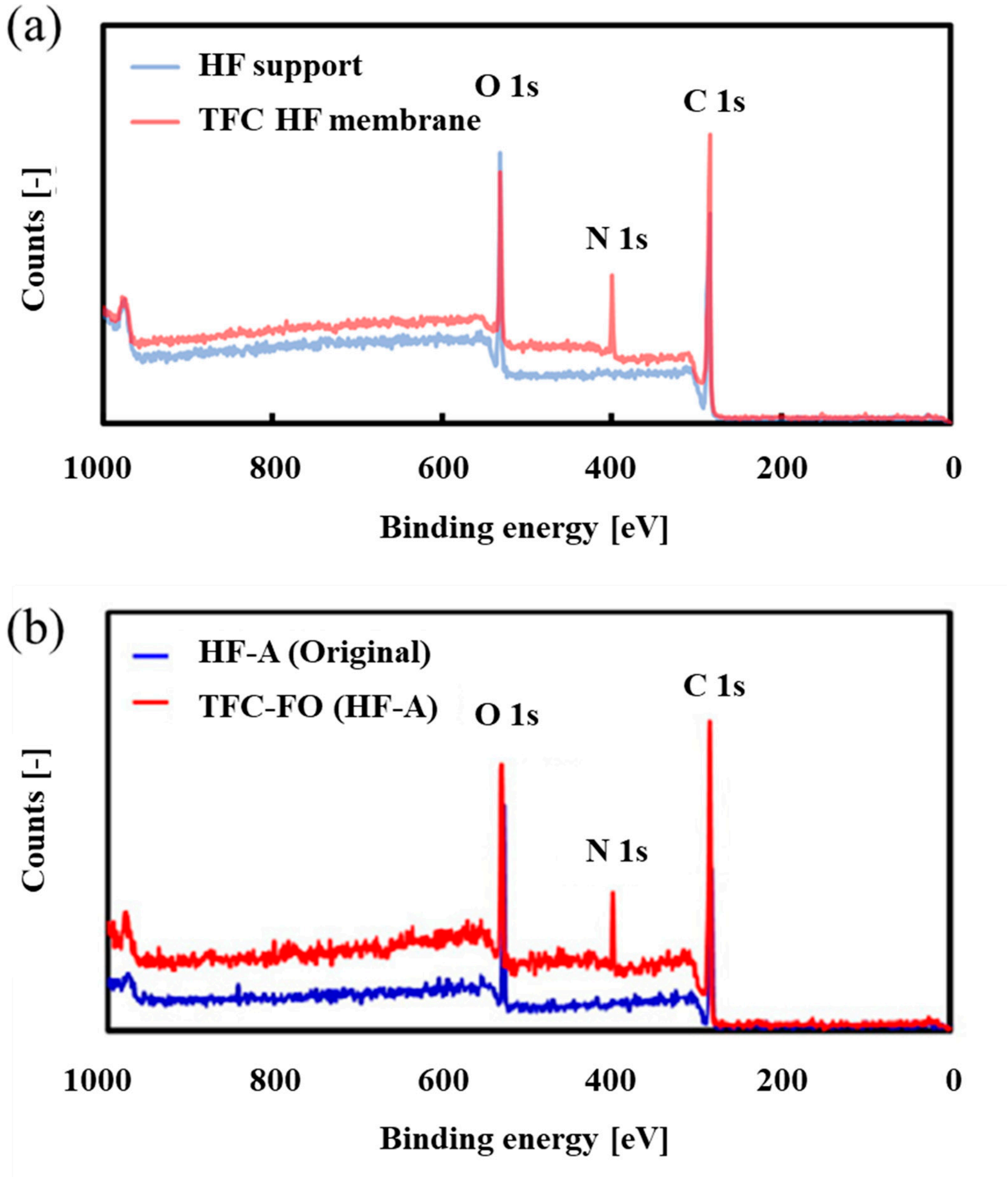

Figure S2. (a) X-ray photoelectron spectra of the bore surface before (HF support) and after (TFC-HF membrane) the interfacial polymerization (this work).
(b) X-ray photoelectron spectra of the shell surface before (HF-A) and after (TFC-FO) the interfacial polymerization in the literature [2].

References

[1] Testing Methods for Bubble Point of Membrane Filters, JIS K3832.
[2] M. Shibuya, M. Yasukawa, S. Mishima, Y. Tanaka, T. Takahashi, H. Matsuyama, A thin-film composite-hollow fiber forward osmosis membrane with a polyketone hollow fiber membrane as a support, *Desalination* **2017**, *402* 33–41. https://doi.org/10.1016/j.desal.2016.09.022.
